# Supplementary figures and images for: The clinicopathology and survival characteristics of patients with POLE proofreading mutations in endometrial carcinoma: A systematic review and meta-analysis
Source: PLoS One. 2022 Feb 9;17(2):e0263585. doi: 10.1371/journal.pone.0263585 (PMC8827442; doi:10.1371/journal.pone.0263585)

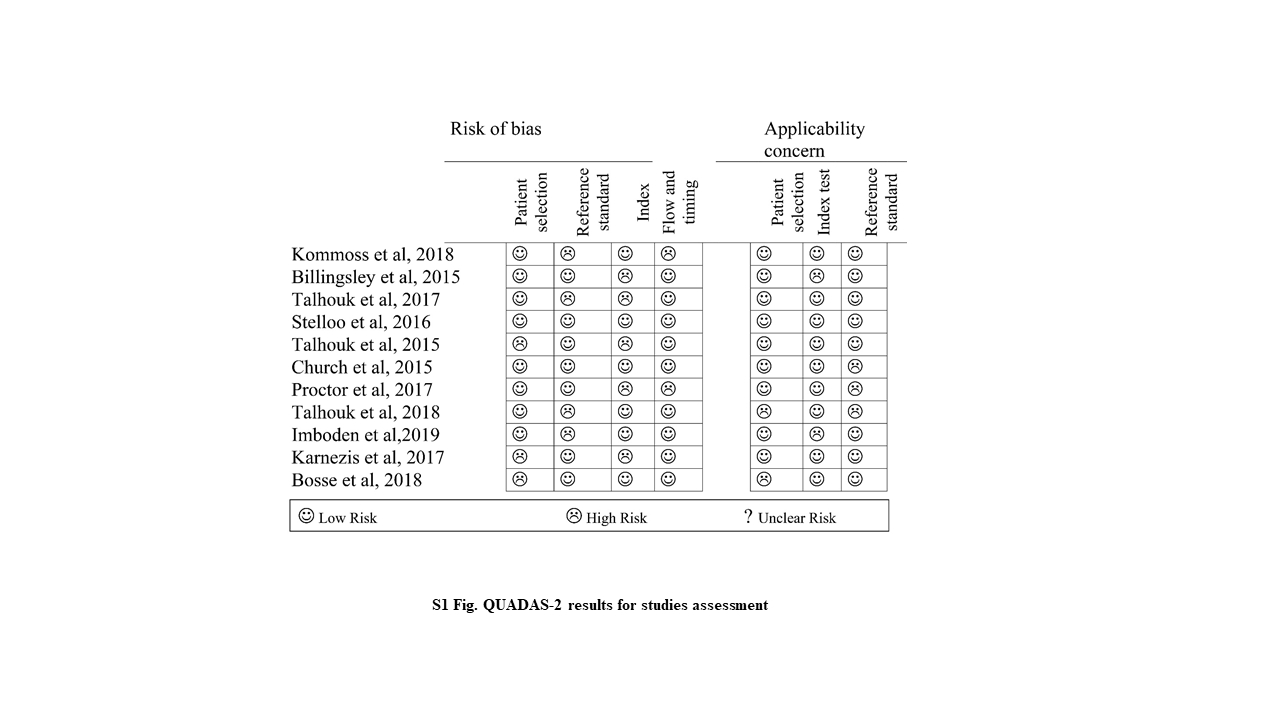

Supplement: S1 Fig — (TIF) [file pone.0263585.s003.tif]

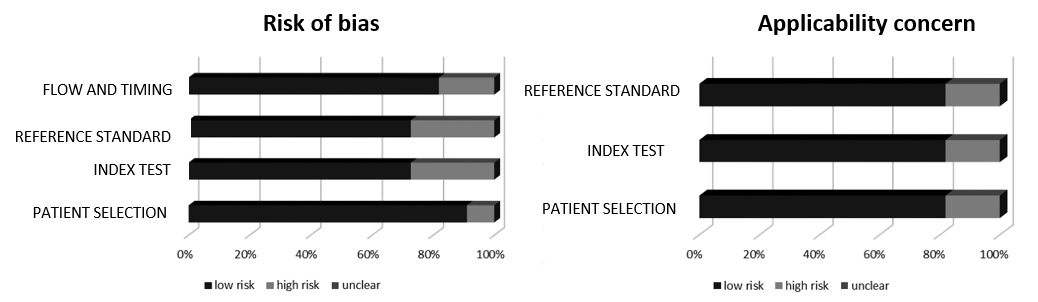

Supplement: S2 Fig — (PNG) [file pone.0263585.s004.png]
