# Supplementary material for: The clinicopathology and survival characteristics of patients with POLE proofreading mutations in endometrial carcinoma: A systematic review and meta-analysis
Source: PLoS One. 2022 Feb 9;17(2):e0263585. doi: 10.1371/journal.pone.0263585 (PMC8827442; doi:10.1371/journal.pone.0263585)
Supplement: S3 Fig — A, pooled proportion of stage I-II. B, pooled proportion of stage III-IV. C, odds ratio of stage I-II POLE mutant EC to stage I-II wild type POLE EC. D, odds ratio of stage III-IV POLE mutant EC to stage III-I VS wild type POLE EC. (DOCX) [file pone.0263585.s005.docx]

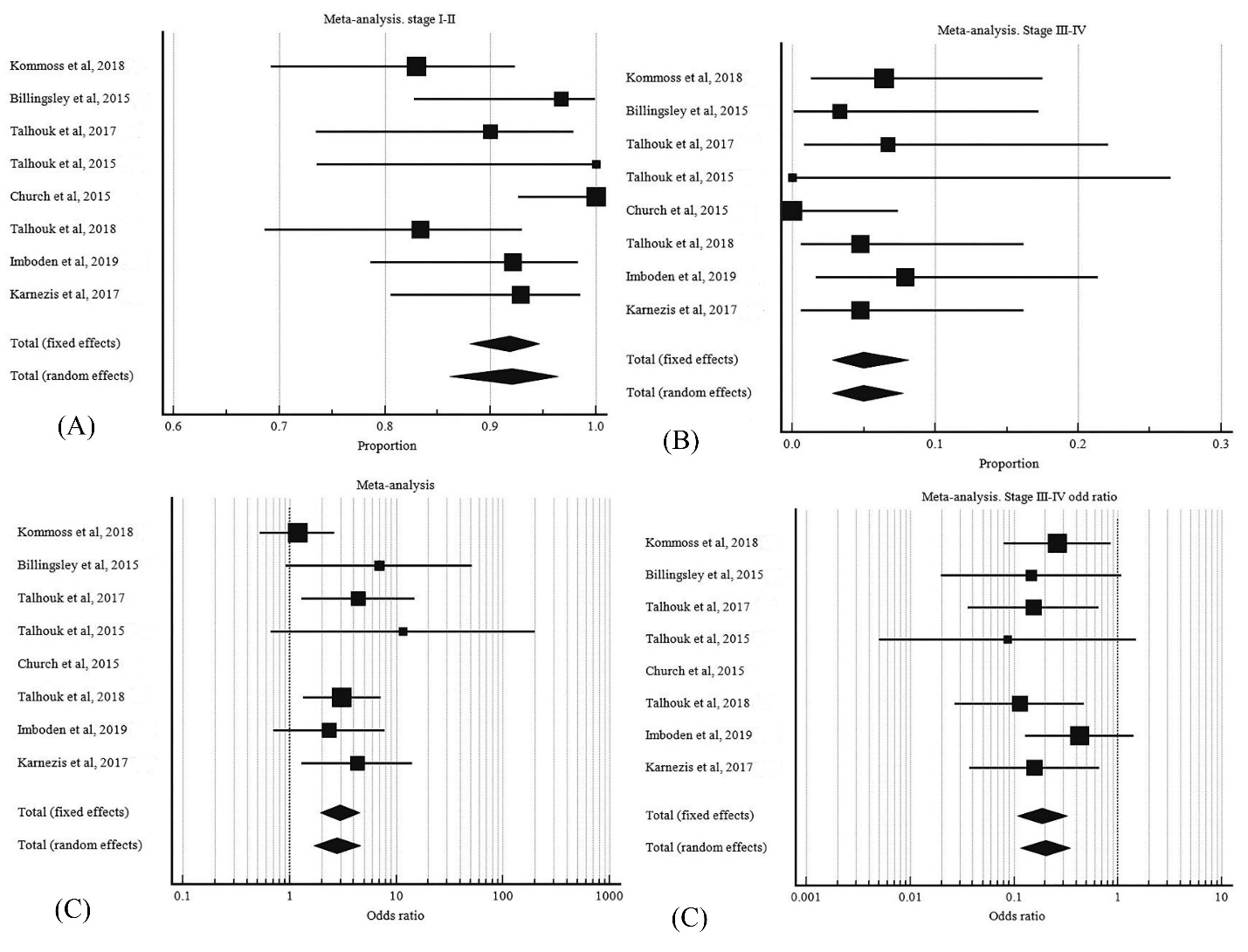


(D)

**S3 Fig**. **FIGO stage in POLE mutant EC.** A, pooled proportion of stage I-II. B, pooled proportion of stage III-IV. **C**, odd ratio of stage I-II POLE mutant EC to stage I-II wild type POLE EC. **D**, odd ratio of stage III-IV POLE mutant EC to stage III-IV wild type POLE EC.
