# Supplementary material for: The clinicopathology and survival characteristics of patients with POLE proofreading mutations in endometrial carcinoma: A systematic review and meta-analysis
Source: PLoS One. 2022 Feb 9;17(2):e0263585. doi: 10.1371/journal.pone.0263585 (PMC8827442; doi:10.1371/journal.pone.0263585)
Supplement: S4 Fig — A, pooled proportion of grade I-II. B, pooled proportion of grade III. C, odds ratio of grade I-II POLE mutant EC to grade I-II wild type POLE EC. D, odds ratio of grade III POLE mutant EC to grade III wild type POLE EC. (DOCX) [file pone.0263585.s006.docx]

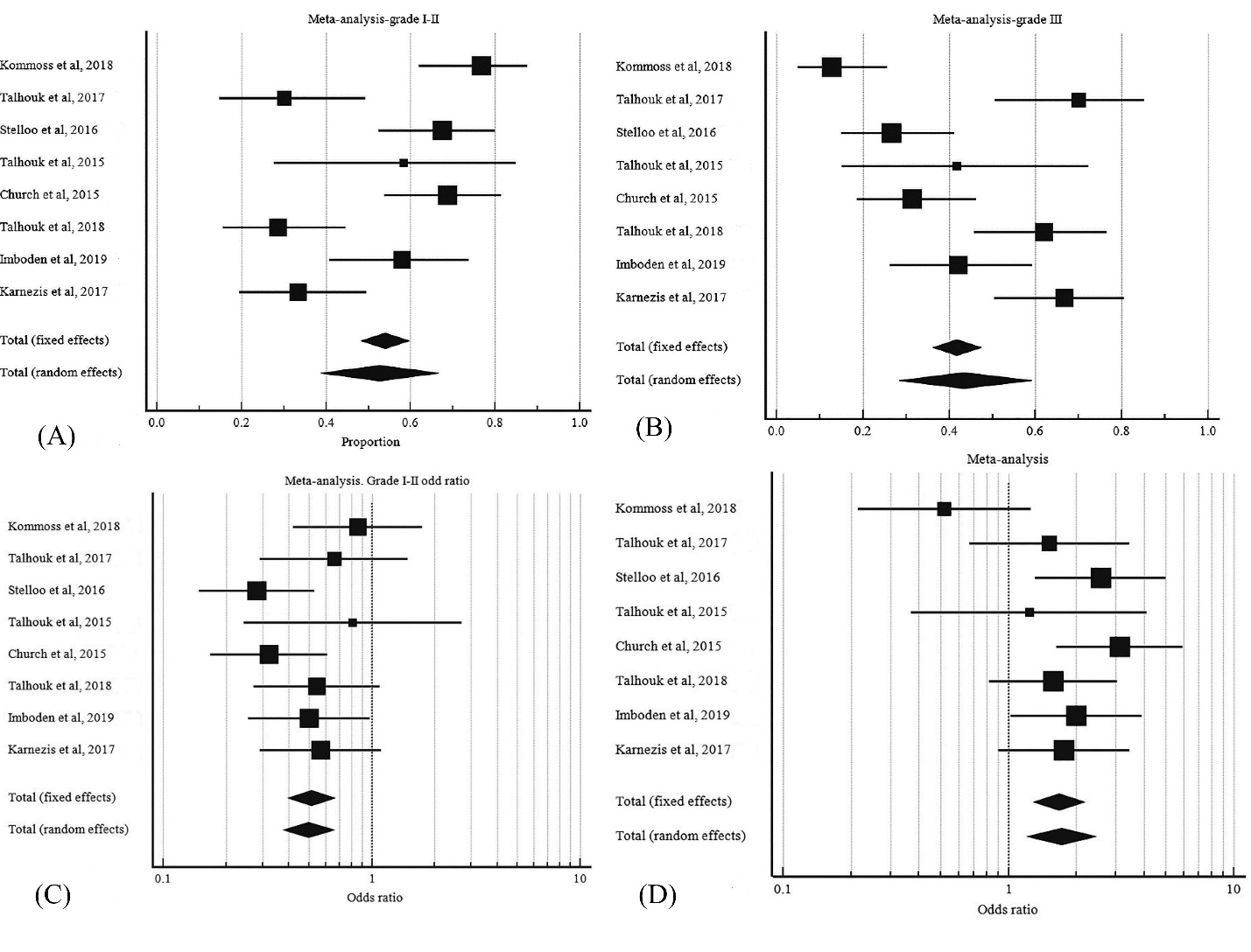


**S4 Fig.** **FIGO grade in POLE mutant EC**. A, pooled proportion of grade I-II. **B**, pooled proportion of grade III. **C**, odd ratio of grade I-II POLE mutant EC to grade I-II wild type POLE EC. **D**, odd ratio of grade III POLE mutant EC to grade III wild type POLE EC.
