# Supplementary material for: The clinicopathology and survival characteristics of patients with POLE proofreading mutations in endometrial carcinoma: A systematic review and meta-analysis
Source: PLoS One. 2022 Feb 9;17(2):e0263585. doi: 10.1371/journal.pone.0263585 (PMC8827442; doi:10.1371/journal.pone.0263585)
Supplement: S5 Fig — A, pooled proportion MI<50%. B, pooled proportion of MI>50%. C, odds ratio of MI<50%. POLE mutant EC to MI<50% wild type POLE EC. D, odds ratio of MI>50% POLE mutant EC to MI>50% wild type POLE EC. (DOCX) [file pone.0263585.s007.docx]

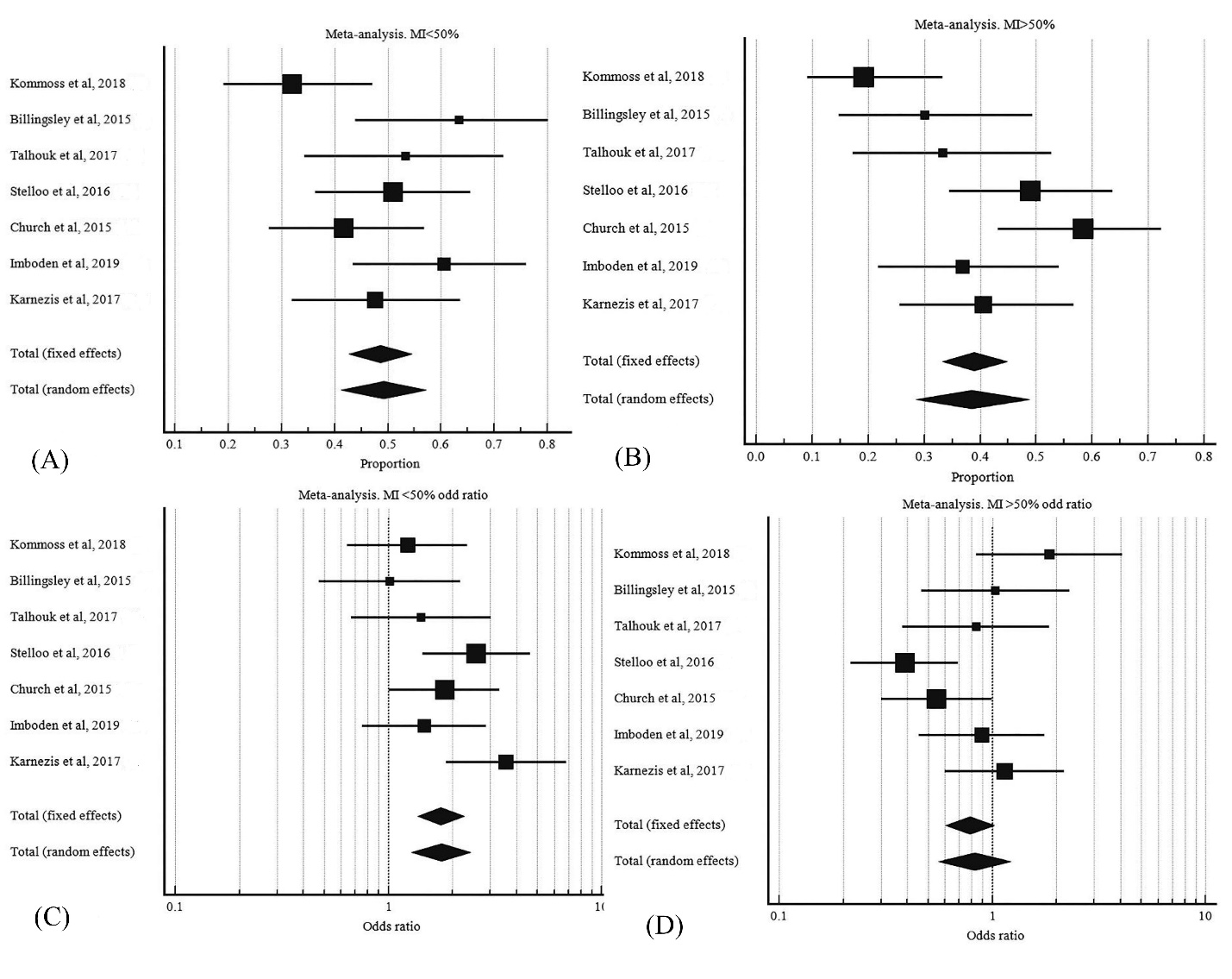


**S5 Fig.** **Myometrial invasion extent in POLE mutant EC.** A, pooled proportion MI<50%. **B**, pooled proportion of MI>50%. **C**, odd ratio of MI<50%. POLE mutant EC to MI<50% wild type POLE EC. **D**, odd ratio of MI>50% POLE mutant EC to MI>50% wild type POLE EC.
