# Supplementary material for: The clinicopathology and survival characteristics of patients with POLE proofreading mutations in endometrial carcinoma: A systematic review and meta-analysis
Source: PLoS One. 2022 Feb 9;17(2):e0263585. doi: 10.1371/journal.pone.0263585 (PMC8827442; doi:10.1371/journal.pone.0263585)
Supplement: S6 Fig — A, pooled proportion LVI. B, odds ratio of LVI POLE mutant EC to LVI wild type POLE EC. (DOCX) [file pone.0263585.s008.docx]

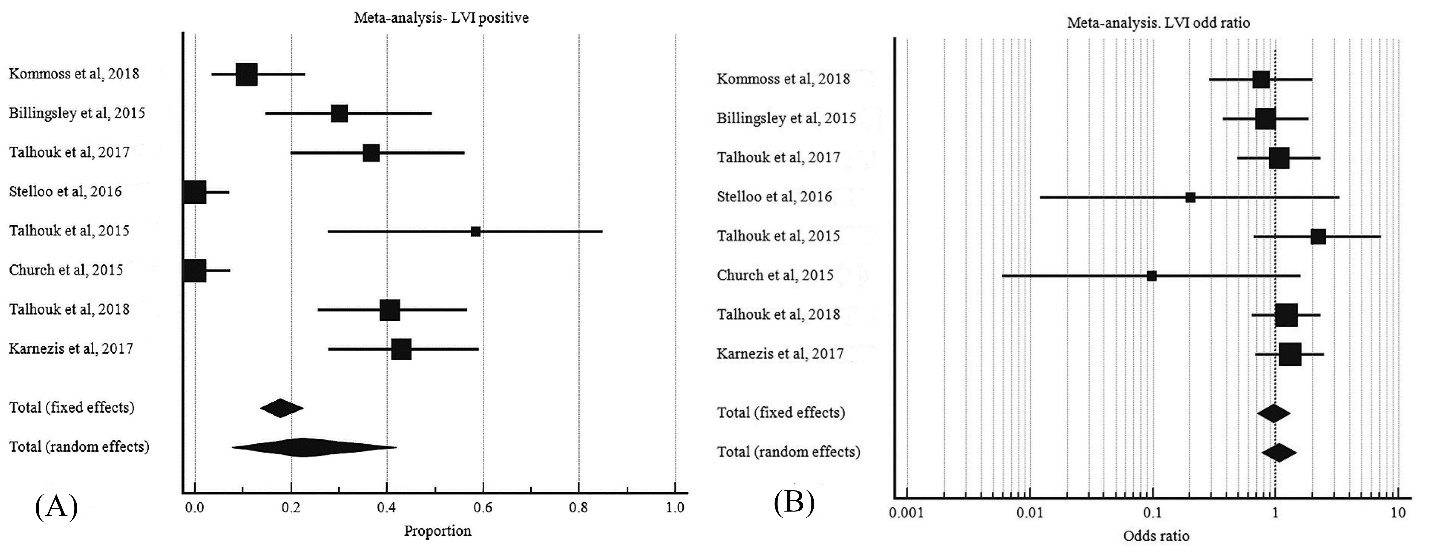


**S6 Fig.** **LVI in POLE mutant EC.** **A**, pooled proportion LVI. **B**, odd ratio of LVI POLE mutant EC to LVI wild type POLE EC.
