# Supplementary material for: The clinicopathology and survival characteristics of patients with POLE proofreading mutations in endometrial carcinoma: A systematic review and meta-analysis
Source: PLoS One. 2022 Feb 9;17(2):e0263585. doi: 10.1371/journal.pone.0263585 (PMC8827442; doi:10.1371/journal.pone.0263585)
Supplement: S7 Fig — A, pooled proportion LN negative B, pooled proportion of LN positive. C, odds ratio of LN negative POLE mutant EC to LN negative wild type POLE EC. D, odds ratio of LN positive POLE mutant EC to LN positive wild type POLE EC. (DOCX) [file pone.0263585.s009.docx]

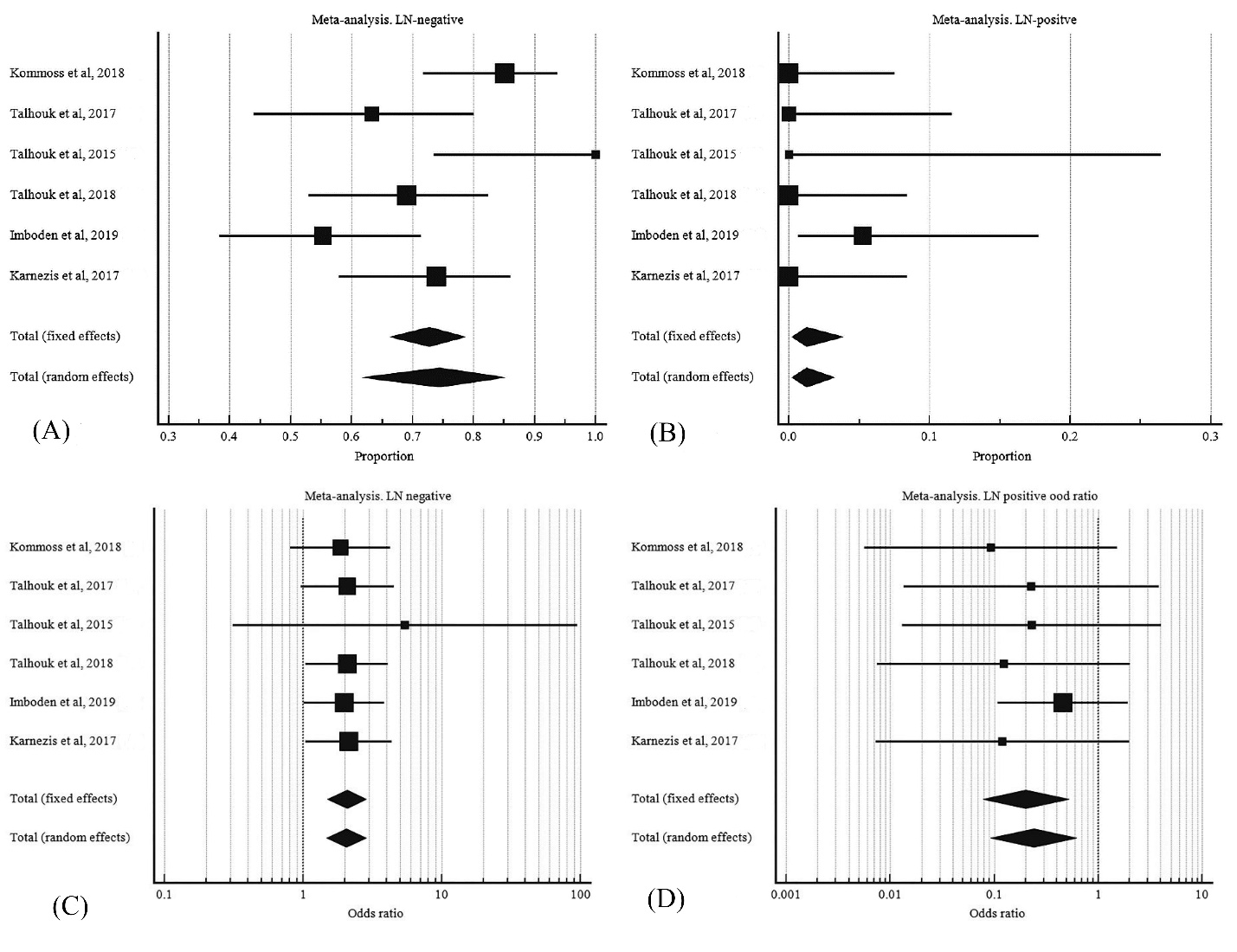


**S7 Fig.** **LN involvements in POLE mutant EC.** **A**, pooled proportion LN negative **B**, pooled proportion of LN positive. **C**, odd ratio of LN negative POLE mutant EC to LN negative wild type POLE EC. **D**, odd ratio of LN positive POLE mutant EC to LN positive wild type POLE EC.
