# Supplementary material for: The clinicopathology and survival characteristics of patients with POLE proofreading mutations in endometrial carcinoma: A systematic review and meta-analysis
Source: PLoS One. 2022 Feb 9;17(2):e0263585. doi: 10.1371/journal.pone.0263585 (PMC8827442; doi:10.1371/journal.pone.0263585)
Supplement: S8 Fig — A, pooled proportion of endometroid type POLE mutant EC B, pooled proportion of non-endometroid type POLE mutant EC. C, odds ratio of endometrioid POLE mutant EC to endometrioid wild type POLE EC. D, odds ratio of non-endometrioid POLE mutant EC to non-endometrioid wild type POLE EC. (DOCX) [file pone.0263585.s010.docx]

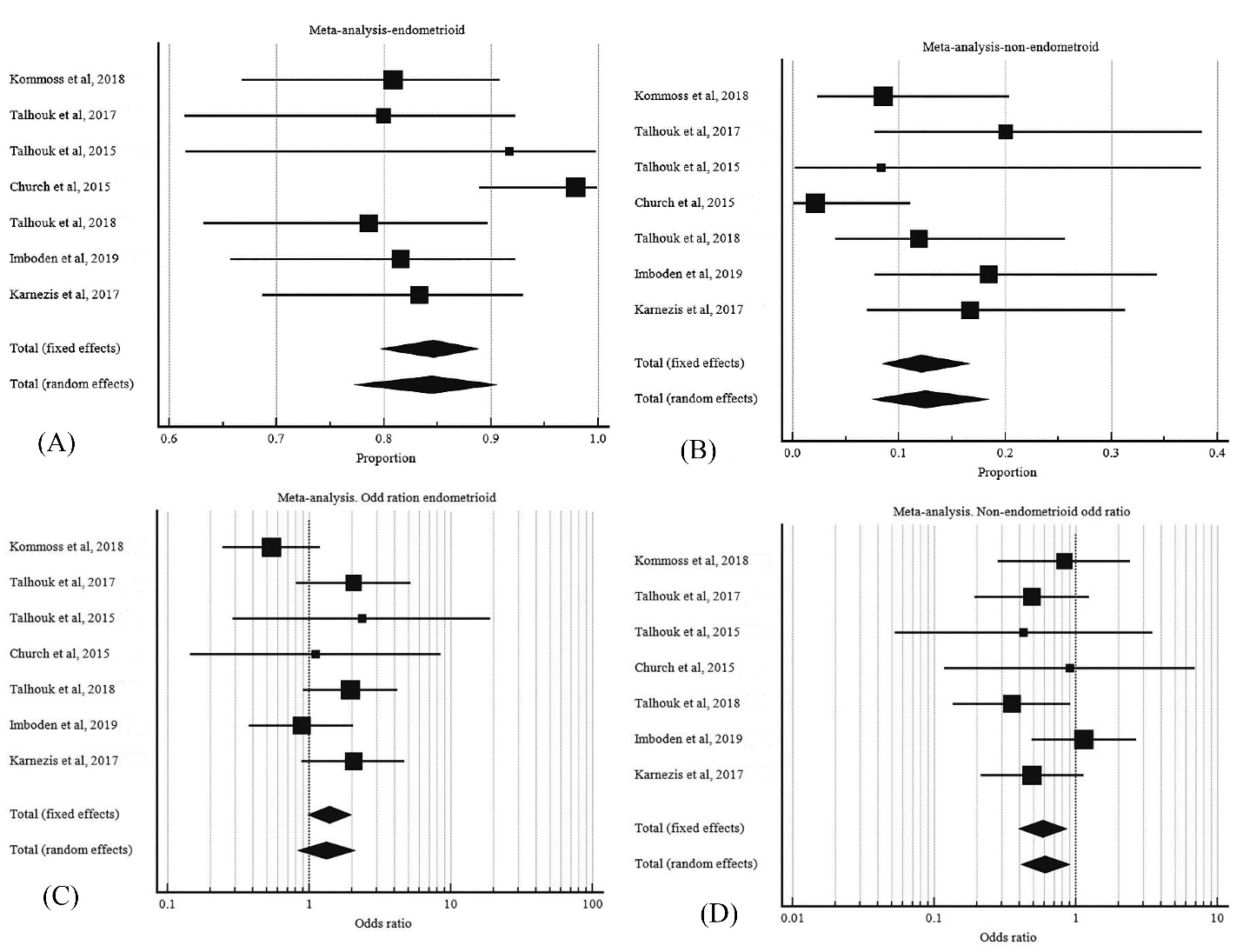


**S8 Fig. Histology of POLE mutant EC**. **A**, pooled proportion of endometroid type POLE mutant EC **B**, pooled proportion of non-endometroid type POLE mutant EC. **C**, odd ratio of endometrioid POLE mutant EC to endometrioid wild type POLE EC. **D**, odd ratio of non-endometrioid POLE mutant EC to non-endometrioid wild type POLE EC.
