# Supplementary material for: The clinicopathology and survival characteristics of patients with POLE proofreading mutations in endometrial carcinoma: A systematic review and meta-analysis
Source: PLoS One. 2022 Feb 9;17(2):e0263585. doi: 10.1371/journal.pone.0263585 (PMC8827442; doi:10.1371/journal.pone.0263585)
Supplement: S1 Table — (DOCX) [file pone.0263585.s011.docx]

**S1 Table. Sensitivity analysis of POLE mutant EC**.

| **Clinicopathological characteristics in EC** | **Total number of patients** | **Pooled % portion**  **(95% CI)** | **I^2^ (95% CI)** | **P-value** |
| --- | --- | --- | --- | --- |
| All studies | 5508 | 8.526 (7.143 to 10.018) | 71.740% (49.330 to 84.240) | 0.001 |
| Omitting Kommoss et al, 2018 | 5056 | 8.345 (6.917 to 9.893) | 71.580% (47.590 to 84.590) | 0.001 |
| Omitting Billingsley et al, 2015 | 4973 | 8.835 (7.379 to 10.409) | 70.630 % (45.580 to 84.150) | 0.002 |
| Omitting Talhouk et al, 2017 | 5189 | 8.456 (6.990 to 10.048) | 73.610 % (51.880 to 85.530) | < 0.001 |
| Omitting Stelloo et al, 2016 | 4674 | 8.837 (7.377 to 10.415) | 69.280 % (42.700 to 83.530) | 0.003 |
| Omitting Talhouk et al, 2015 | 5365 | 8.530 (7.077 to 10.104) | 74.250 % (53.220 to 85.820) | < 0.001 |
| Omitting Church et al, 2015 | 4720 | 8.816 (7.327 to 10.429) | 70.800 % (45.940 to 84.230) | 0.002 |
| Omitting Proctor et al, 2017 | 5418 | 8.234 (6.922 to 9.649) | 69.880 % (43.980 to 83.810) | 0.003 |
| Omitting Talhouk et al, 2018 | 5048 | 8.481 (6.989 to 10.102) | 73.630 % (51.930 to 85.540) | < 0.001 |
| Omitting Imboden et al, 2019 | 4909 | 8.777 (7.269 to 10.412) | 72.600 % (49.740 to 85.060) | 0.001 |
| Omitting Karnezis et al, 2017 | 5048 | 8.481 (6.989 to 10.102) | 73.630 % (51.930 to 85.540) | < 0.001 |
| Omitting Bosse et al, 2018 | 5132 | 8.103 (6.842 to 9.461) | 64.790 % (32.970 to 81.500) | 0.001 |
| Omitting Kommoss et al, 2018 | 5056 | 8.464 (6.980 to 10.077) | 73.460 % (51.570 to 85.460) | < 0.001 |
