# Supplementary material for: The clinicopathology and survival characteristics of patients with POLE proofreading mutations in endometrial carcinoma: A systematic review and meta-analysis
Source: PLoS One. 2022 Feb 9;17(2):e0263585. doi: 10.1371/journal.pone.0263585 (PMC8827442; doi:10.1371/journal.pone.0263585)
Supplement: S2 Table — (DOCX) [file pone.0263585.s012.docx]

**S2 Table. Sensitivity analysis for cases involved in the analysis of overall survival.**

| Studies with overall survival (OS) | Estimated hazard ratio (HR) (95%CI) | P-value | I^2^ (95% CI) | P-value  for I^2^ | Model used |
| --- | --- | --- | --- | --- | --- |
| All cases | 0.772 (0.574 to 1.039) | 0.088 | 0.000% (0.000 to 50.920) | 0.621 | Fixed effect |
| Omit Kommoss et al, 2018 | 0.761 (0.556 to 1.041) | 0.088 | 0.000% (0.000 to 57.830) | 0.535 | Fixed effect |
| Omit Billingsley et al, 2015 | 0.830 (0.611 to 1.129) | 0.235 | 0.000% (0.000 to 29.180) | 0.855 | Fixed effect |
| Omit Talhouk et al, 2017 | 0.760 (0.559 to 1.032) | 0.078 | 0.000% (0.000 to 57.220) | 0.546 | Fixed effect |
| Omit Stelloo et al, 2016 | 0.748 (0.549 to 1.020) | 0.066 | 0.000% (0.000 to 55.480) | 0.578 | Fixed effect |
| Omit Talhouk et al 2015 | 0.790 (0.586 to 1.065) | 0.122 | 0.000% (0.000 to 48.890) | 0.679 | Fixed effect |
| Omit Church et al, 2015 | 0.694 (0.492 to 0.978) | 0.037 | 0.000% (0.000 to 48.900) | 0.679 | Fixed effect |
| Omit Proctor et al, 2017 | 0.762 (0.565 to 1.027) | 0.075 | 0.000% (0.000 to 54.960) | 0.587 | Fixed effect |
| Omit Talhouk et al, 2018 | 0.767 (0.565 to 1.041) | 0.089 | 0.000% (0.000 to 58.070) | 0.530 | Fixed effect |
| Omit Karnezis et al, 2017 | 0.793 (0.581 to 1.082) | 0.143 | 0.000% (0.000 to 56.630) | 0.557 | Fixed effect |
| Omit Bosse et al, 2018 | 0.824 (0.595 to 1.141) | 0.244 | 0.000% (0.000 to 52.970) | 0.620 | Fixed effect |
| Omit Kommoss et al, 2018 | 0.766 (0.561 to 1.046) | 0.094 | 0.000% (0.000 to 58.100) | 0.530 | Fixed effect |
