# Supplementary material for: The clinicopathology and survival characteristics of patients with POLE proofreading mutations in endometrial carcinoma: A systematic review and meta-analysis
Source: PLoS One. 2022 Feb 9;17(2):e0263585. doi: 10.1371/journal.pone.0263585 (PMC8827442; doi:10.1371/journal.pone.0263585)
Supplement: S3 Table — (DOCX) [file pone.0263585.s013.docx]

**S3 Table. Sensitivity analysis of disease specific survival studies**

| Studies with disease specific survival (DSS) | Estimated hazard ratio (HR) (95%CI) | P-value | I^2^ (95% CI) | P-value  for I^2^ | Model used |
| --- | --- | --- | --- | --- | --- |
| Omit Kommoss et al, 2018 | 0.404 (0.302 to 0.541) | <0.001 | 0.000% (0.000 to 0.000) | 0.979 | Fixed effect |
| Omit Talhouk et al, 2017 | 0.354 (0.178 to 0.704) | 0.003 | 0.000% (0.000 to 0.000) | 0.982 | Fixed effect |
| Omit Talhouk et al 2015 | 0.413 (0.309 to 0.551) | <0.001 | 0.000% (0.000 to 0.000) | 0.990 | Fixed effect |
| Omit Church et al, 2015 | 0.415 (0.310 to 0.554) | <0.001 | 0.000% (0.000 to 0.000) | 0.993 | Fixed effect |
| Omit Proctor et al, 2017 | 0.406 (0.304 to 0.541) | <0.001 | 0.000% (0.000 to 0.000) | 0.980 | Fixed effect |
| Omit Talhouk et al, 2018 | 0.409 (0.306 to 0.546) | <0.001 | 0.000% (0.000 to 0.000) | 0.975 | Fixed effect |
| Omit Imboden et al, 2019 | 0.412 (0.308 to 0.550) | <0.001 | 0.000% (0.000 to 0.000) | 0.982 | Fixed effect |
| Omit Karnezis et al, 2017 | 0.404 (0.301 to 0.542) | <0.001 | 0.000% (0.000 to 0.000) | 0.977 | Fixed effect |
| Omit Kommoss et al, 2018 | 0.409 (0.306 to 0.547) | <0.001 | 0.000% (0.000 to 0.000) | 0.976 | Fixed effect |
