# Supplementary material for: The clinicopathology and survival characteristics of patients with POLE proofreading mutations in endometrial carcinoma: A systematic review and meta-analysis
Source: PLoS One. 2022 Feb 9;17(2):e0263585. doi: 10.1371/journal.pone.0263585 (PMC8827442; doi:10.1371/journal.pone.0263585)
Supplement: S4 Table — (DOCX) [file pone.0263585.s014.docx]

**S4 Table. Sensitivity analysis of studies involved in the analysis of progression free survival**

| Studies with overall survival (OS) | Estimated hazard ratio (HR) (95%CI) | P-value | I^2^ (95% CI) | P-value  for I^2^ | Model |
| --- | --- | --- | --- | --- | --- |
| All cases | 0.231 (0.117 to 0.456) | <0.001 | 0.000% (0.000 to 0.000) | 0.963 | Fixed effect |
| Omit Kommoss et al, 2018 | 0.206 (0.0988 to 0.428) | <0.001 | 0.000% (0.000 to 0.000) | 0.989 | Fixed effect |
| Omit Billingsley et al, 2015 | 0.233 (0.113 to 0.480) | <0.001 | 0.000% (0.000 to 20.860) | 0.911 | Fixed effect |
| Omit Talhouk et al, 2018 | 0.247 (0.118 to 0.515) | <0.001 | 0.000% (0.000 to 1.360) | 0.939 | Fixed effect |
| Omit Imboden et al, 2019 | 0.246 (0.119 to 0.507) | <0.001 | 0.00% (0.000 to 0.000) | 0.945 | Fixed effect |
| Omit Karnezis et al, 2017 | 0.226 (0.108 to 0.473) | <0.001 | 0.000% (0.000 to 19.420) | 0.914 | Fixed effect |
| Omit Bosse et al, 2018 | 0.232 (0.102 to 0.529) | 0.001 | 0.000% (0.000 to 21.070) | 0.911 | Fixed effect |
