# Supplementary material for: The clinicopathology and survival characteristics of patients with POLE proofreading mutations in endometrial carcinoma: A systematic review and meta-analysis
Source: PLoS One. 2022 Feb 9;17(2):e0263585. doi: 10.1371/journal.pone.0263585 (PMC8827442; doi:10.1371/journal.pone.0263585)
Supplement: Attachment — Submitted filename: answer to editor (1).docx [file pone.0263585.s016.docx]

1. **Please describe the rationale for carrying out this meta-analysis in the context of current knowledge in the field. Please indicate whether there are previous meta-analyses in the literature which covered this research question (or closely related ones) and if that is the case, list the relevant reference(s) and comment on how the current meta-analysis differs from the previously published reports.**

***Answer:*** We believe the content of the paper is significant: (1) our systematic review and meta-analysis study has resolved the reported clinicopathology variations of POLE-mutant endometrial carcinoma. Indeed, we have confirmed the (2) prognostic benefit of the POLE (exonuclease domain mutant) subtype by analysis of an expanded cohort of studies of survival - presently the largest number of patient cases. The previous published meta-analysis cohort that most closely covers this research question included only six studies concerned with the survival of endometrial carcinoma patients with POLE mutant tumors [7]. The present meta-analysis also (3) reports additional clinicopathology parameters not discussed in the prior study [7]. This present meta-analysis with an expanded cohort number increases the robustness of results for interpretation. Our study is key to understanding the improved prognostic potential of the POLE mutant subtype and highlighting the most pertinent clinicopathology features for the benefit of both clinicians and researchers alike.

REFERENCE: 7.Jumaah AS, Al-Haddad HS, Mahdi LH, McAllister KA, Yasseen AA. The frequency of POLE-mutation in endometrial carcinoma and prognostic implications: A systemic review and meta-analysis. JPTM. 2020. 25. Doi: https://doi.org/10.4132/jptm.2020.07.2

2. **Please provide the full details of the databases searched to identify the studies included in this meta-analysis**.

***Answer:***

Search strategy:

The internet literature review searched PubMed, web of science, and Embase. Furthermore, all oncology and pathology journals available in Scimago websites were searched. There was no cut-off date applied to the study. The following search terms were applied: “endometrial carcinoma”, “endometrial cancer”, “POLE EDM mutations”, “EC”, “POLE mutant,”, “clinical characteristics” and “prognosis”. All references in the included studies were screened for potentially suitable published articles. There were no language restrictions, however fortunately all eligible studies were published in English. (material and method; lines 80-89).

3. **Please clarify whether there are previous genome-wide association studies (GWAS) relevant to the research question covered by the meta-analysis and whether these were included in the analysis. If previous related GWAS are available, please indicate whether the results of this meta-analysis are consistent with the findings of the GWAS publications, and if this is not the case, please provide an explanation for this discrepancy.**

Answer: After meticulous internet searches - we could not found previous genome-wide association studies (GWAS) relevant to the research question covered by the meta-analysis

4. In addition, please complete the following checklist and upload it as a Supplementary file when you resubmit your manuscript.  The checklist can be downloaded here: <http://www.plos.org/wp-content/uploads/2013/05/meta-analysis-on-genetic-association-studies-form.docx>.

Answer: Check list is provided.

5. ) Please confirm that you have included all items recommended in the PRISMA checklist including:

   - the full electronic search strategy used to identify studies with all search terms and limits for at least one database.
   -  the search inclusion dates
   - an assessment of publication bias using graphical methods (e.g. Funnel plot) and statistical methods (e.g. Egger’s test) as appropriate

Answer: done. A full PRISMA check list had been attached as supplementary materials table.
